# Supplementary figures and images for: DNA Barcoding Study of Representative Thymus Species in Bulgaria
Source: Plants (Basel). 2022 Jan 20;11(3):270. doi: 10.3390/plants11030270 (PMC8840554; doi:10.3390/plants11030270)

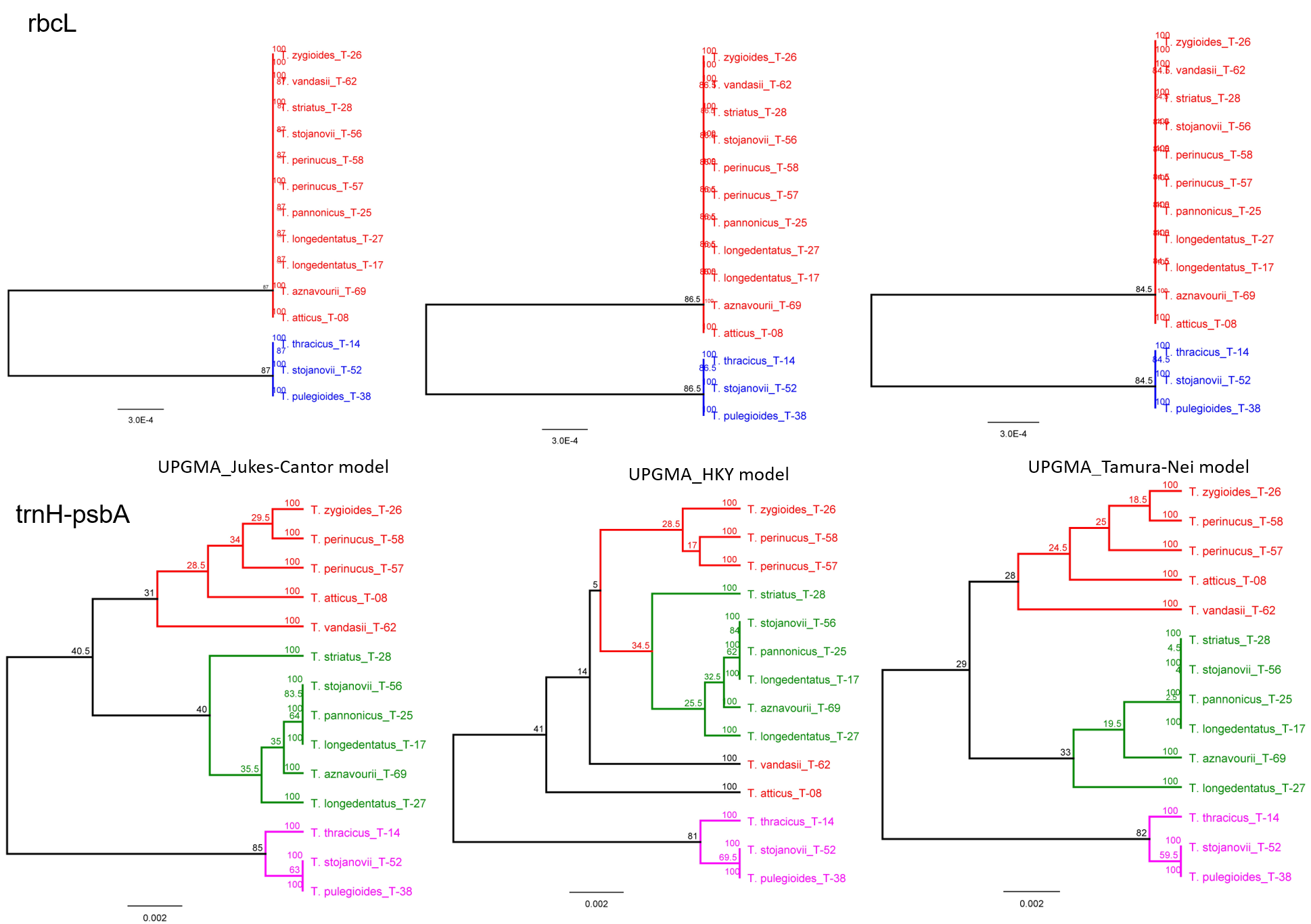

Supplement: Supplementary file 1 [file plants-11-00270-s001.zip › Suppl. Fig. 1 trees rbcL_trnH-psbA.jpeg]

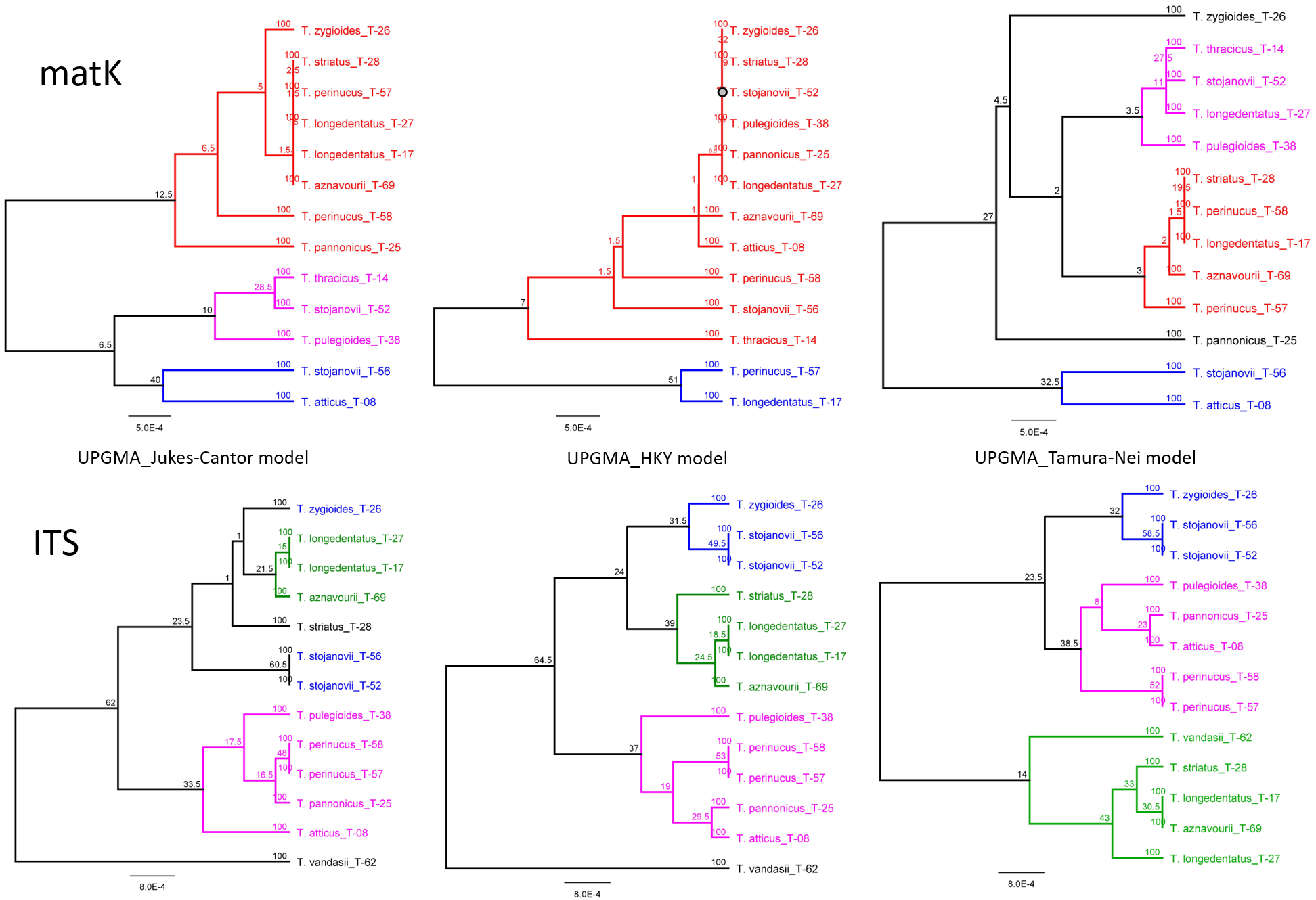

Supplement: Supplementary file 1 [file plants-11-00270-s001.zip › Suppl. Fig. 2 trees matK_ITS.jpeg]
